# Using Augmented Reality Technology to Optimize Transfacet Lumbar Interbody Fusion: A Case Report

**DOI:** 10.3390/jcm13051513

**Published:** 2024-03-06

**Authors:** Anas Bardeesi, Troy Q. Tabarestani, Stephen M. Bergin, Chuan-Ching Huang, Christopher I. Shaffrey, Walter F. Wiggins, Muhammad M. Abd-El-Barr

**Affiliations:** 1 Department of Neurosurgery, Duke University Hospital, Durham, NC 27710, USA; 2 Duke University School of Medicine, Durham, NC 27710, USA; 3 Department of Radiology, Duke University Hospital, Durham, NC 27710, USA

**Keywords:** transfacet MIS TLIF, augmented reality, segmentation technology

## Abstract

The transfacet minimally invasive transforaminal lumbar interbody fusion (MIS-TLIF) is a novel approach available for the management of lumbar spondylolisthesis. It avoids the need to manipulate either of the exiting or traversing nerve roots, both protected by the bony boundaries of the approach. With the advancement in operative technologies such as navigation, mapping, segmentation, and augmented reality (AR), surgeons are prompted to utilize these technologies to enhance their surgical outcomes. A 36-year-old male patient was complaining of chronic progressive lower back pain. He was found to have grade 2 L4/5 spondylolisthesis. We studied the feasibility of a trans-Kambin or a transfacet MIS-TLIF, and decided to proceed with the latter given the wider corridor it provides. Preoperative trajectory planning and level segmentation in addition to intraoperative navigation and image merging were all utilized to provide an AR model to guide us through the surgery. The use of AR can build on the safety and learning of novel surgical approaches to spine pathologies. However, larger high-quality studies are needed to further objectively analyze its impact on surgical outcomes and to expand on its application.

## 1. Introduction

With new augmented reality (AR) headsets, automatic cortical pathway segmentations, and navigation techniques, the field of neurosurgery will soon be deeply intertwined with advancing segmentation technologies [1]. However, in the current literature, the implementation of these technologies specifically for spine surgery has been rather limited to vertebrae visualization and pedicle screw placement [2,3,4,5]. These same technologies can be used for other aspects of minimally invasive spine surgery, most specifically, interbody fusions [6]. As documented throughout the literature, minimally invasive techniques have relied primarily on accessing the disc space through several key anatomic corridors [7,8]. These include Kambin’s Triangle, which at times necessitates a facetectomy to increase the area of cannula placement, or the ‘safe triangle’ method which requires exiting nerve root (ENR) retraction rostrally [9]. Subsequently, there is a risk for post-operative ipsilateral radiculopathies due to ENR irritation or damage with instrument manipulation or portal docking [10]. Likewise, recent studies have highlighted the importance of visualizing these corridors prior to surgery since the majority of triangles will not permit the necessary cannula diameter for discectomy or adequate cage placement [11]. Even though multiple studies have demonstrated the necessity of pre-operatively measuring these corridors in order to define the safest trajectory for each patient’s specific spinal anatomy, a surgeon must also be prepared to alter their operative course if those corridors will not provide enough room for a successful interbody fusion [12,13]. For that reason, other minimally invasive approaches, such as the relatively novel transfacet approach, have begun to gain popularity as alternatives routes to the disc space since they provide better working areas for endplate preparation and larger cage dimensions [14,15]. Having said this, it is difficult to compare the relative areas and trajectories of each approach from only examining pre-operative 2D images like CT and MRI alone. To fully assess the safety of each and to define the most accurate trajectory avoiding all key neurovascular structures, a 3D model with pre-operative radiographic analysis and intra-operative navigational assistance is needed. This case illustrates an example of how novel segmentation technology aided in each phase of an interbody fusion: determining which anatomic corridor provided the safest access to the disc space, outlining the key structures to guide intra-operative planning, implementing image fusion to account for positioning changes, and using AR imaging to overlay traditional microscopic imaging and segmentation imaging to allow for safe intraoperative transfacet MIS-TLIF.

## 2. Methodology

### 2.1. Patient History and Physical Examination

**Figure 1:**
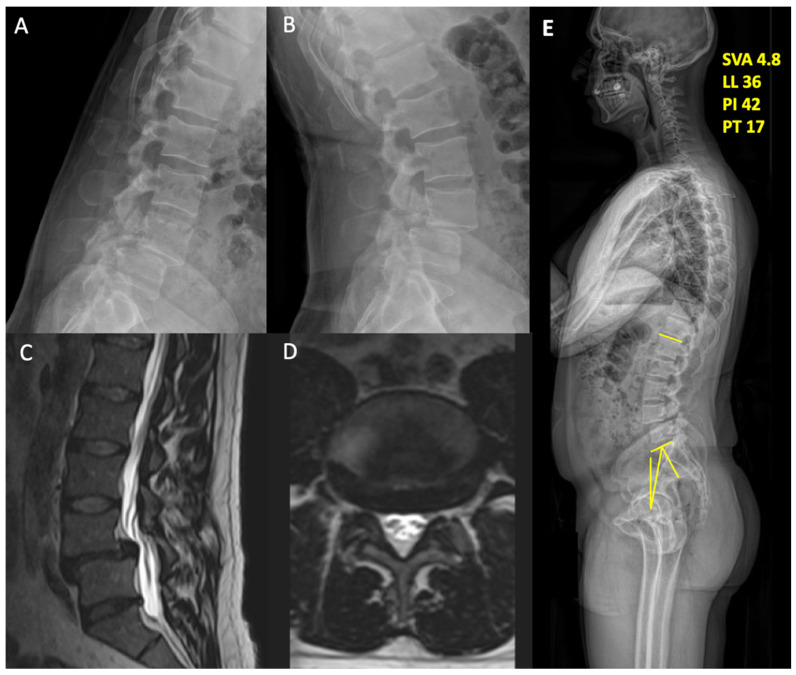
(**A**) Flexion and (**B**) extension views of standing lumbar x-rays, demonstrating the pars defect and the L4/5 grade 2 spondylolesthesis. (**C**) Sagittal and (**D**) axial cuts of T2 MRI showing the bilateral lateral recess and foraminal stenosis. (**E**) Lateral whole-spine film demonstrating the adequate sagittal and spinopelvic balance preoperatively.

The patient is a 36-year-old male with no pertinent past medical history with a chief complaint of worsening lower back pain that began 7 years ago. On initial presentation, he stated that his pain was very severe, causing him to make constant modifications to his everyday life. He was unable to walk for long periods without having to rest. He also trialed conservative therapy including physical therapy, traction, and a weight loss program, reaching a BMI of 28.25 kg/m^2^, all of which did not provide him with adequate relief. His neurological exam was non-focal. On imaging, his flexion/extension lumbar spine radiographs showed bilateral L4 pars interarticularis fractures, and his MRI demonstrated a grade 2 L4/5 spondylolisthesis as well as a small central disc at L5/S1 (Figure 1). His pre-operative visual analogue scale (VAS) for back pain was 7, and the patient-reported outcomes measurement information system (PROMIS) scores were as follows: PROMIS pain—61 (moderate) and PROMIS function—39 (moderate dysfunction). His Oswestry Disability Index (ODI) score was 14. Given that his symptoms progressed despite therapy, he decided to have surgery. Informed consent was acquired before the procedure.

We involved the patient in the discussion of the appropriate fusion approach. Since his preoperative parameters were not suggestive of sagittal imbalance or a PI-LL mismatch, a single-level fusion surgery was proposed. The anterior lumbar interbody fusion (ALIF) was technically difficult since the iliac vessels were branching off in front of the L3/4 level. The lateral and oblique lumbar interbody fusions (LLIF and OLIF) were possible feasible approaches; however, the patient did not feel comfortable with either, as he was young and preferred not having any anterolateral incisions from a cosmetic point of view. Thus, we planned a posterior approach. To decide which posterior-only approach to use, we used preoperative segmentation.

### 2.2. Kambin’s Triangle and TransFacet Segmentation

Three-dimensional isotropic T2-weighted MRI sequences acquired at 1 mm slice thickness were used during the preoperative planning phase. First, the caudal/rostral vertebrae, thecal sac, and disc were all segmented with the Smartbrush 2.5feature in BrainLab (BrainLab, Munich, Germany). Then, the individual nerve roots (exiting and traversing) were identified in the axial, coronal, and sagittal planes. Manual segmentation was carried out using a region-growing algorithm. The Align feature was used to orient the images in a direction that maximized the cross-sectional area of Kambin’s triangle and the transfacet corridor. BrainLab then generated a 3D representation of the structures to visualize spatial proximity. Since the ENR and TNR had already been segmented, it helped ensure that we were not overlapping the outlined corridor with any parts of the neural anatomy. Given that there have not been any prior radiographic papers defining the 3D boundaries of the transfacet approach, we defined the corridor as the TNR extending until the caudal pedicle as the base, the theca as the height, and the ENR as the hypotenuse. A board-certified neuroradiologist and neurosurgeon confirmed each segmentation.

### 2.3. Pre-Operative Measurements

**Figure 2:**
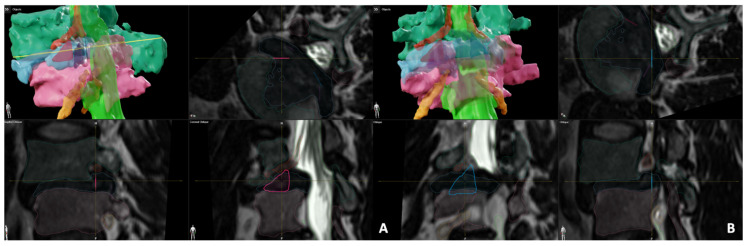
Preoperative segmentation of (**A**) the left Kambin’s triangle (red triangle) with an area of 100.6 mm^2^, and (**B**) the right transfacet corridor (blue triangle) with an area of 135.8 mm^2^.

Once each approach was visualized bilaterally at the operative level, the measurement functions in the BrainLab software were used to calculate the area of each corridor. For both the transfacet and Kambin’s Triangle, the largest triangle approximation method was used with the formula: 0.5 × base × height (mm^2^). Next, the largest possible cannula diameter size was found for each approach using BrainLab’s circle measurement function, ensuring the planned cannula did not extend past the boundaries of either segmented corridor. To further verify the accuracy of the measurements, another formula was used to calculate the maximum permissible diameter that would fit within the segmented triangles: height + base − hypotenuse. If there was a discrepancy between the two values for cannula diameter, the average number was used. The largest Kambin’s area was the left-sided L4/5 of 100.6 mm^2^, with a diameter of 8 mm compared to the largest transfacet area, which was 135.8 mm^2^ on the right side, with an 11 mm diameter (Figure 2).

**Figure 3:**
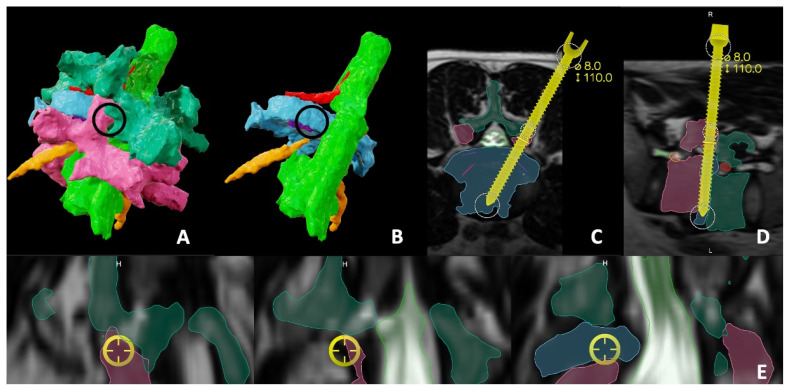
(**A**) A 3D model of the segmented L4-5 demonstrating L4 vertebra (dark green), L5 vertebra (pink), thecal sac (light green), disc space (Blue) exiting nerve root (red), traversing nerve root (yellow) and the working channel for a transfacet approach (circle). (**B**) The same model, after simulated removal of the L4 and L5 vertebrae to show the clear corridor to the disc space. Axial (**C**) and sagittal (**D**) preoperative planning of the approach to the interbody space modeled by an elongated screw layered over the segmented 3D MRI. (**E**) Sequential bird’s eye views of the selected trajectory confirming its safety in avoiding the nerves.

Therefore, taking into consideration the measurements we had for both the Kambin’s and transfacet areas, we decided to proceed with a right-sided transfacet minimally invasive (MIS) transforaminal lumbar interbody fusion (TLIF) (Figure 3).

### 2.4. Intra-Operative Details

**Figure 4:**
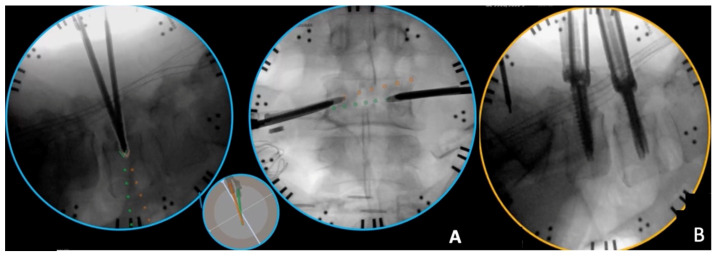
Intraoperative fluoroscopy-based tracking that was utilized for the different stages of percutaneous pedicle screw insertion. (**A**) Bilateral simultaneous advancement of both jamshidi needles into the L4 pedicles. Each of the dots is equal to 10 mm. This allows us to know where the jamshidi needle is in both dimensions, and where its anticipated location will be if advanced. (**B**) Final view of the pedicle screws at both levels.

**Figure 5:**
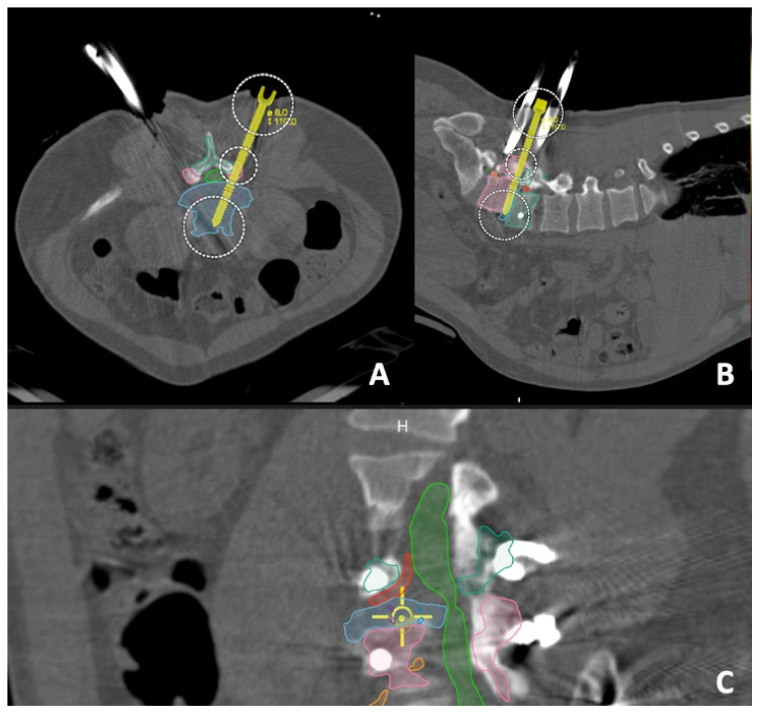
(**A**) Axial, (**B**) sagittal and (**C**) bird’s eye views of the merging of the preoperative planning with the intraoperative CT scan assuring the accuracy and confirming the safety of our corridor, modelled by an elongated screw, before advancing with the steps of interbody fusion.

The patient underwent general anesthesia with erector spinae plane (ESP) block with liposomal bupivacaine (Exparel, Parsippany, NJ, USA). Neuromonitoring was used for motor-evoked potentials (MEPs), somatosensory-evoked potentials (SSEPs) and electromyography (EMG). The patient was flipped prone onto a Jackson bed. Instrument-tracking fluoroscopy (Track X, Hillsborough, NC, USA), which is a fluoroscopy-based tracking system, was utilized to plan our incisions, which were two 1-inch incisions about 3 cm paramedian on both sides. We then started with percutaneous pedicle screw placement for the L4 and L5 levels simultaneously on each side (Figure 4). All screws were stimulated above 20 mA. We then placed a reference marker in the posterior superior iliac spine (PSIS), and then a CT was performed to ensure accurate placement of the pedicle screws. The intraoperative CT and preoperative MRI with the segmentation were merged and then fused with the preoperative planning into a model that was projected on the operating microscope through Brainlab^®^ Spine Curvature Correction Software version 1 (Figure 5). The accuracy of this software was examined previously and was reported to be less than 1.34 mm of median error, after fusing pre-operative MRI with intraoperative CT images [16]. 

**Figure 6:**
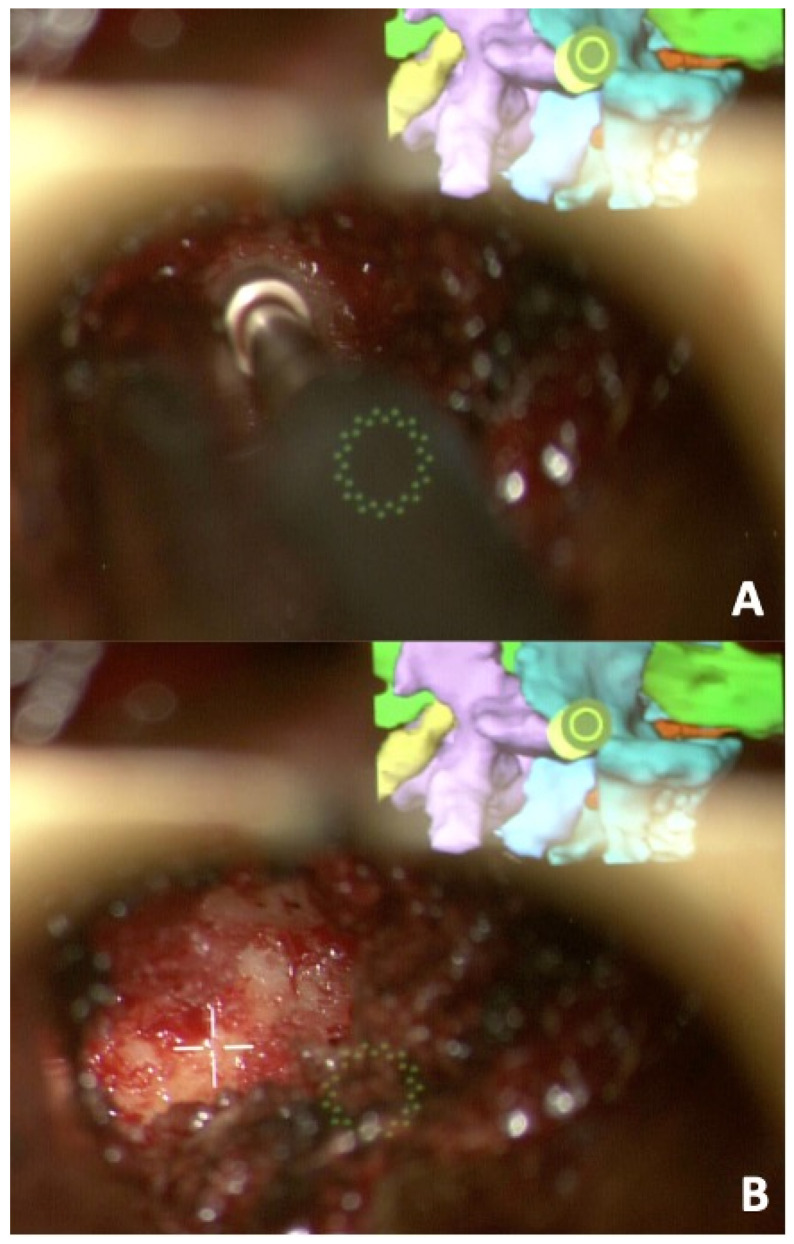
(**A**,**B**) The microscopic views during the drilling phase with the projected AR model guiding the surgeon on the top right corner. The planned cannula in yellow assures our drilling is in keeping with the planned trajectory.

**Figure 7:**
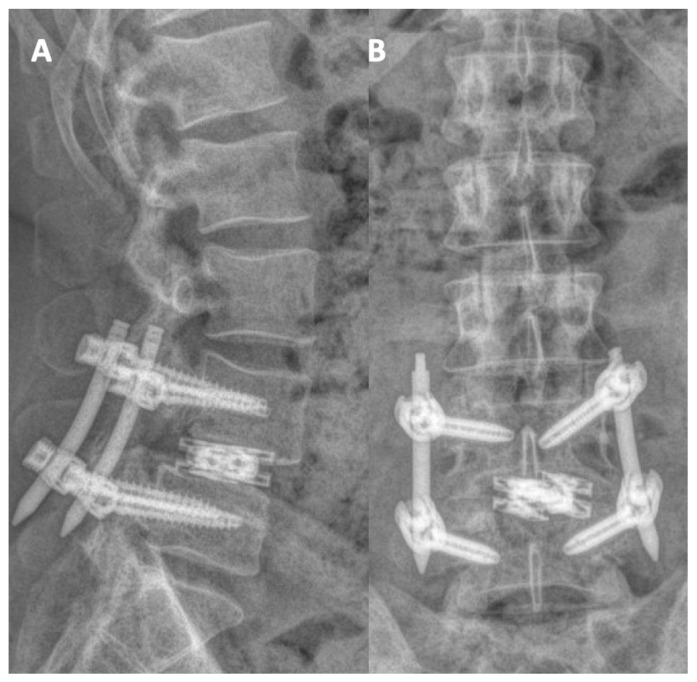
(**A**) AP and (**B**) lateral views of the postoperative standing X-rays demonstrating satisfactory reduction of the slip and reassuring hardware integrity.

We then used our right-sided incision to dock our tubes on the L4-5 facet joint. We were able to switch between a plain and an AR-projected view on the microscope (Figure 6). Using careful drilling, we started drilling initially through the medial aspect of the superior articulating facet then into the facet joint, staying within the bony confinement of the facet joint. Once the disc space was reached, discectomy ensued with shavers and curettes, and we eventually were able to advance a Dual-X (Amplify Surgical, Irvine, CA, USA) cage that was expanded to 10 mm in height and 21 mm in width. The cage and space were filled with autograft and allograft. We then denuded and drilled the L4-5 facets on the contralateral side with placement of allograft and autograft for posterolateral fusions. Bilateral rods were placed, and final fluoroscopic images assured the integrity of the construct, followed by standard closure of fascia and skin. The procedure was completed in 3 h, the estimated blood loss was 100 mL, and there were no perioperative complications. The patient was discharged home on his second postoperative day with reassuring postoperative standing films. (Figure 7). In his early postoperative visits, he was reporting significant improvement of his lower back pain, and he was resuming his usual activities with no concerns. Six months after the surgery, he was able to resume all his regular activities. His back pain VAS was 1, ODI was 0, and PROMIS pain and function scores were 39 and 64, respectively (within normal limits).

## 3. Discussion

Like the rest of the surgical specialties, spine surgery has been incorporating a diverse variety of advanced technology that aims to enhance surgical outcomes, trainees’ education, and patients’ safety. The field of extended reality, which is the umbrella term that collectively refers to augmented reality (AR), mixed reality (MR) and virtual reality (VR), is among those technologies currently being utilized in the field of spine surgery. While VR, where the entire environment and all tools are virtual, and MR, where there is interaction between the physical and virtual worlds, are currently implemented in educational and simulation purposes for medical students and surgical trainees, AR may have the most direct effect on patient care [17,18]. AR can be defined as having computer-generated information layered over a real-world object. The use of AR in spine surgery has been trending in the literature, with its use adding to the safety and efficiency of open and MIS pedicle screw insertion [19] or in conjunction with intraoperative navigation [20,21,22]. We also found two studies when AR was used for both microscopic [23] and endoscopic TLIF [24]. The two possible AR operating systems currently available on the market are either the head-mounted display (HMD) or systems that incorporate into the operating microscope, with the latter being the system used in our case.

Image guidance and navigation systems have been utilized by surgeons to maximize the safety of traversing through small anatomic corridors during either the planning or crucial part of a procedure. Furthermore, having a 3D representation of the origin or course of major anatomical structures builds on that aim. In our review, we noted that nerve segmentation technology, whether manually or in an automated fashion, was applied for mapping of peripheral nerves, similar to trigeminal nerve segmentation and its relation to vascular structures and brainstem [25] or for the facial nerve as reference during mastoidectomy [26]. Nerve segmentation was also reported for predicting and avoiding exiting nerve root injury within the confinement of Kambin’s triangle for endoscopic discectomy [11,27,28] or for percutaneous interbody fusions [12]. In this report, we aimed to implement both AR and 3D segmentation of all structures, including the exiting nerve root to guide us through safer completion of our L4-5 transfacet TLIF.

The novel transfacet MIS-TLIF has been recently gaining more attention; however, there remains a paucity of articles addressing its use and outcomes. One study of 68 patients who underwent transfacet MIS-TLIF with expandable cages found significant clinical improvements and restoration of global sagittal segmental parameters as well as regional alignment correction in patients with hyperlordosis [23]. Another study has looked at an endoscopic transfacet approach for 41 patients, with improvement in ODI and VAS scores [29]. The transfacet TLIF requires less bony resection than the traditional TLIF. The transfacet TLIF preserves the medial inferior articular process, lateral superior articular process, and rostral pars, which protects the traversing and exiting nerve roots. This is advantageous compared to the standard TLIF, which exposes these nerve roots during surgery. Although it might be argued that identifying and retracting the TNR during a standard TLIF is more reassuring for the operating surgeon, the consequences associated with doing so are not forgiving, with direct root injury during exposure or cage placement or irritation with post op deficits by simply retracting it. The lack of preoperative radiculopathy, such as in this case, is also a reason against needing to directly decompress the nerve root and adding unnecessary risk to the procedure. Additionally, the transfacet TLIF provides an alternative option for cases where the trans-Kambin approach is deemed unsafe with a narrow corridor placing the ENR at risk. One potential disadvantage of the transfacet TLIF is for patients who have an abnormally inferior exiting nerve root, in which case the transfacet TLIF may be more difficult and potentially more dangerous than the traditional TLIF.

Preoperative MRI with parasagittal T2 MRI cuts can be used to ensure the exiting nerve root is not normally inferior in the foramen. Parasagittal T2 MRI can help evaluate foraminal anatomy and Kambin’s triangle, and augmented reality can play an important role in this presurgical evaluation. The transfacet MIS-TLIF leverages a unique bony working corridor to access the disc space for discectomy and interbody placement, and technologies including AR present an opportunity to improve operative efficiencies and patient outcomes. By not only depending on surgeons’ instrumental tactile feedback and anatomical orientation during the bony work, the AR interface together with navigation both supplement that step with additional reassurance until the disc space is reached. Knowing where the ENR was with the help of AR and where it was passing beyond the bones added to the safety of the approach and eliminated the need to find it. Additionally, this whole surgical experience has added a narrative, live demonstration of the key steps of the approach to the knowledge and understanding of the scrubbed surgical team, building on the learning curve of such complex procedures.

As a case report addressing the application of novel techniques to a relatively novel surgical approach, we anticipated multiple limitations to our report that should serve as the basis for larger studies with stronger evidence. Our report, resembling the previously reported series for the MIS transfacet approach, does not compare the surgery and patients’ outcomes of the approach to its counterparts, the standard transforaminal or the trans-Kambin approaches.

## 4. Conclusions

We present a unique case of using augmented reality for preoperative planning, and also intraoperative implementation of a relatively new method of transforaminal lumbar interbody fusion. We show how this technology can help ensure patients’ safety and an excellent outcome. Larger studies focused on the applicability of such technology are needed.

## Data Availability

No new data were created or analyzed in this study. Data sharing is not applicable to this article.
